# Influence of the 2000-m ergometer test on indirect markers of intestinal injury in competitive elite rowers in different training phases

**DOI:** 10.1186/s13102-023-00761-w

**Published:** 2023-11-07

**Authors:** Hanna Dziewiecka, Anna Kasperska, Joanna Ostapiuk–Karolczuk, Justyna Cichoń-Woźniak, Piotr Basta, Anna Skarpańska-Stejnborn

**Affiliations:** 1 Department of Biological Sciences, Faculty of Physical Culture in Gorzów Wielkopolski, Poznań University of Physical Education, Estkowskiego 13, 66-400 Gorzów, Wielkopolski, Poland; 2 Department of Physical Education and Sport, Faculty of Physical Culture in Gorzów Wielkopolski, Poznań University of Physical Education, Estkowskiego 13, 66-400 Gorzów, Wielkopolski, Poland

**Keywords:** Athletes, Rowers, Gut injury, I-FABP, Zonulin, LPS, LBP, Il-6, Preparation phase

## Abstract

**Background:**

We examined the effect of the 2000-m ergometer test on gut injury in competitive elite rowers in two different training phases. Given that inflammatory markers during the competitive phase are higher, we hypothesise that markers of intestinal injury are also more elevated during that phase.

**Methods:**

We performed this study during the preparatory phase (Test I) and competitive phase (Test II) of annual training. We included 10 competitive elite rowers, members of the Polish Rowing Team, in the study after applying the inclusion/exclusion criteria. The participants performed a 2000-m ergometer test during both phases (Tests I and II). We collected blood samples before the test, immediately after the test and after 1 h of recovery. We measured the levels of interleukin 6 (IL-6), intestinal fatty acid binding protein (I-FABP), lipopolysaccharide (LPS), lipopolysaccharide-binding protein (LBP), and zonulin.

**Results:**

There were no significant changes over time in Test I and Test II in the gut integrity markers. There were significantly lower I-FABP and IL-6 levels after the test for Test II compared with Test I. The pre-test LPS level was significantly lower for Test II compared with Test I. The pre-test LBP and zonulin levels were numerically lower in Test II, but the differences were not significant.

**Conclusions:**

The 2000-m ergometer test showed no influence on gut integrity markers. However, there were differences in the response to exercise between Tests I and II. The lower level of gut injury markers after extreme exercise tests carried out during the preparation period may be the result of adaptive mechanisms and could indicate that rationally conducted training significantly decreases intestinal injury.

## Introduction

Exercise-associated gastrointestinal symptoms (GIS) are common among endurance athletes and can vary in severity from minor discomfort to clinically significant signs [1]. GIS such as nausea and diarrhoea, flatulence, the urge to regurgitate, heartburn, or even blood in the stool during exercise may occur and minimally or substantially negatively impact exercise performance, resulting in reduced workload, cessation of exercise, and/or withdrawal from activity (1). Exercise-induced gastrointestinal syndrome leads to GIS during exercise. Two pathways promote it – circulatory–gastrointestinal and neuroendocrine–gastrointestinal – but a potential third factor in the form of mechanical strain may also occur (i.e., body position and/or the mechanical strain on the splanchnic arena) [1]. Local intestinal ischaemia is one of the main physiological factors that cause cell damage and dysfunction through reduced adenosine triphosphate (ATP) synthesis from mitochondrial respiration. There may be changes involving Paneth cells (specialised antimicrobial protein-secreting cells), goblet cells (mucus-producing cells), and the tight junction proteins (claudin and occludin) that prevent the infiltration of pathogenic organisms into the systemic circulation. These changes may cause leakage of endotoxins such as lipopolysaccharide (LPS) and proinflammatory cytokines through the epithelial wall [2]. At the same time, gastrointestinal functions including motility, digestion, and absorption may be reduced due to epithelial injury and/or dysfunction. Moreover, deactivation of the submucosa, the myenteric plexus, or other components of the gastrointestinal tract that are affected by exercise may be responsible for impaired gastrointestinal function [3]. This functional impairment may partly explain the reduced absorption of intestinal nutrients observed after strenuous exercise [4].

In systematic reviews, researchers have reported that the minimum threshold of exercise stress necessary to evoke GIS is ≥ 2 h at 60% maximal oxygen uptake (V̇O_2max_) at an ambient temperature of ≥ 35.0 °C or ≥ 3 h at 60% V̇O_2max_ at temperate conditions in laboratory-controlled studies [1, 3, 5]. The authors also assumed that anything less than the above conditions is insufficient to elicit gastrointestinal integrity that may present on a clinical level [1, 4, 5]. In contrast, Aune et al. [6] investigated the impact of a bout of strenuous exercise on gut leakage markers in patients with suspected coronary artery diseases (N = 287). The mean exercise duration was 9.31 min, and the authors reported a significant increase in LPS and lipopolysaccharide-binding protein (LBP) [6]. Edwards et al. [7] suggested that the exercise mode may not be as crucial as its intensity, although the protocol used in that study lasted 45 min [7]. Research on exercise-induced permeability has usually focused on healthy individuals and has used endurance-style protocols [8]. Still, little is known about changes in gut permeability in competitive elite athletes the different phases of the training cycle; of note, each phase differs regarding the work load. During rowing competitions, the teams usually finish the race within seconds (or less) of each other. The incidence and severity of GIS incidence may influence the capacity of the athlete to perform at his or her maximum level [8].

The 2000-m rowing race distance at the Olympic level is covered in approximately 5.5–7.5 min, depending on the boat class, sex, and environmental conditions. Rowing requires a high mechanical power output of 450–550 W (9) at a high percentage of V̇O_2max_. After the race, the blood lactic acid (LA) concentration can reach 15–16 mmol/l [10]. Elite athletes spend about 100–210 min a day training [11]. This training comprises rowing, nonspecific endurance, and strength training. Additionally, rowing training is associated with a unique metabolic demand [9]. Thus, we examined the changes in markers of gut integrity in competitive elite rowers (members of the Polish National Team; we measured the eliteness/expertise of the athletes in accordance with Swan et al. [12]) during the 2000-m ergometer test at two times during the training cycle: the preparatory phase (Test I) and the competitive phase (Test II). Given that inflammatory markers during the competitive phase are higher, we hypothesise that markers of intestinal permeability are also more elevated during that phase [9].

## Materials and methods

### Participants

Eighteen male National Polish Rowing Team members (heavyweight rowers) were recruited, but only 10 met the inclusion criteria and participated in the study; all of the participants finished the two 2000-m ergometer tests. Before each test, the anthropometric parameters were assessed using an electronic scale to the nearest 0.05 kg (Tanita BC-980 MA, Tanita Corporation, Tokyo, Japan). The results are presented in Table 1. The study was performed by following the Declaration of Helsinki. The study protocol was approved by the local Ethics Committee at Poznań University of Medical Sciences (decision no. 314/22 in 2022). All participants were informed of the study procedures and gave their written consent.

**Table 1:** The anthropometric characteristics of the participants (in the morning after an overnight fast before Tests I and II).

	Body fat [%]	Lean body mass [kg]	High [cm]	Body mass [kg]	BMI [kg/m^2^]	Age [years]	Total body water [%]	Training internship [years]
Test I								
Mean	10.1	79.7	190.7	88.2	24.4	20.4	61.14	6.9
SD	2.5	5.3	4.7	7.1	1.3	1.4	2.0	1.5
Test II								
Mean	9.5	78.9	190.7	87.7	24.1	20.4	64.42	6.9
SD	3.0	5.7	4.7	7.3	1.5	1.4	2.2	1.5

SD, standard deviation

### Inclusion criteria

The inclusion criteria were a minimum of 5 years of training, a minimum total training time of 240 min per week, membership in the Polish Rowing Team, and finishing the 2000-m ergometer test.

### Exclusion criteria

The exclusion criteria were antibiotic therapy, probiotics, prebiotics, metformin, a dietary regime, and health problems within the last 3 months.

### Training programme

The training profile, including the intensity, volume (in minutes), and type (specific, i.e., rowing: endurance, speed, technical; and nonspecific: strength, jogging), were noted daily. In addition, the intensity of the training was classified concerning the LA threshold (4 mmol/l): an extensive (below the LA threshold) or an intensive (above the LA threshold) workload (Table 2).

**Table 2:** Training programme before the tests

	Test I (preparatory phase)									Test II (competitive phase)							
Days	1	2	3	4	5	6	7	8		1	2	3	4	5	6	7	8
Time rowed, minutes/day	105	90	30	90	80	90	70	**T** **E** **S** **T**	Time rowed, minutes/day	90	0	70	120	160	80	90	**T** **E** **S** **T**
Distance rowed, km/day	20	18	6	20	18	20	12		Distance rowed, km/day	20	0	12	22	36	18	20	
Training for force development, minutes/day	0	0	90	0	80	0	0		Training for force development, minutes/day	0	0	0	0	0	0	0	
Extensive endurance rowing training time, minutes/day	8	90	30	50	64	90	70		Extensive endurance rowing training time, minutes/day	64	0	70	120	136	80	90	
**High-intensity** endurance rowing training time, minutes/day	20	0	0	40	16	0	0		**Very high-intensity** endurance rowing training time, minutes/day	26	0	0	0	24	0	0	
Nonspecific training (running, etc.), minutes/day	75	10	90	15	55	10	20		Nonspecific training (running, etc.), minutes/day	90	0	10	30	20	10	10	
**Total training time, minutes/day**	**190**	**100**	**210**	**105**	**215**	**100**	**90**		**Total training time, minutes/day**	**180**	**0**	**80**	**150**	**180**	**90**	**100**	

### Food intake

The total dietary intake was analysed by a dietitian before each test by using the 24-hour dietary recall method. The dietician carefully checked each questionnaire and was available for the participants during all meals. Then, the energy, carbohydrate, protein, and fat were measured through the commercially available DietetykPro program (DietetykPro, Wrocław, Poland).

**Fig. 1:**
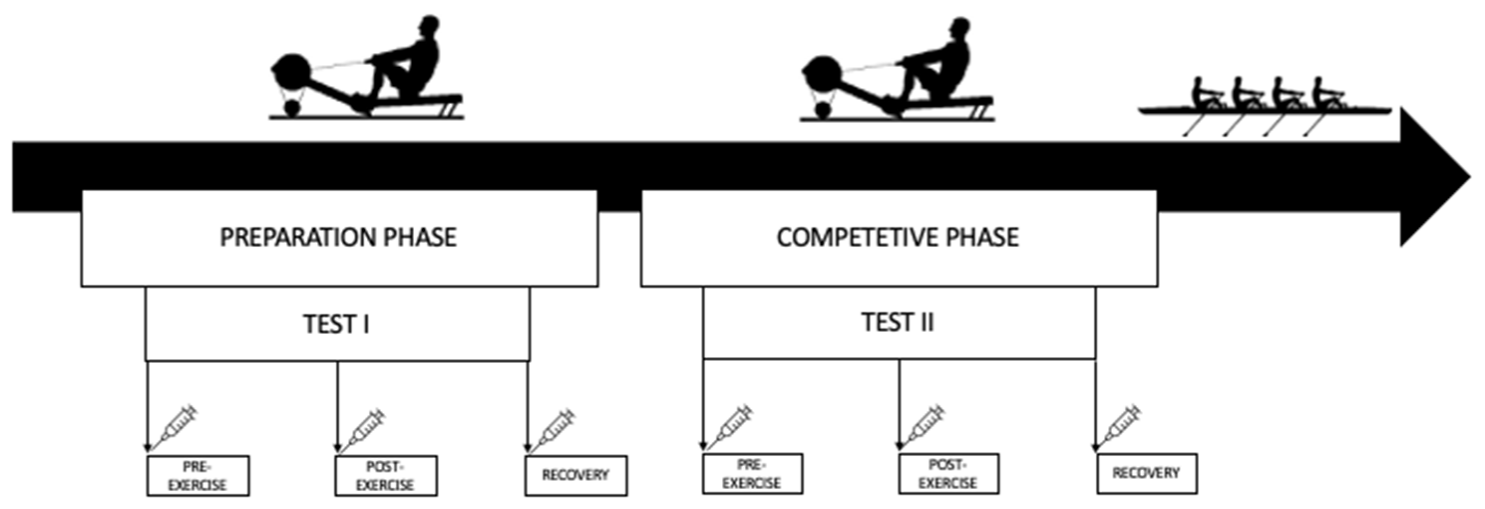
The study design and timeline

### Exercise test

For Tests I and II, the athletes performed a controlled test at a distance of 2000 m (Fig. 1). The break between the tests was nearly 10 weeks (68 days). Test I was performed at the beginning of the preparatory phase, while Test II was performed at the beginning of the competitive phase. The participants rowed a distance of 2000 m on the ergometer (Concept II, USA) as fast as possible because the test results were considered during the selection for the championship team. Hence, the athletes were highly motivated to perform both tests at maximal effort. The exercise test was performed at 10:00 a.m. on each day. Before the test, the participants ate a small, light meal and were hydrated (Table 1). Before the tests, each participant completed a 5-minute individual warm-up.

### Material collection and examination

Samples were collected at the same three-time points: Pre (pre-exercise), after overnight fasting; Post (immediately post-exercise), and Recovery (after 1 h of recovery) for Tests I II.

Blood samples were collected from the antecubital vein into 9-ml polyethylene tubes (to obtain serum) and centrifuged at 3000 rpm for 10 min. The serum was frozen and stored at − 80 °C until analysis. In addition, capillary blood samples were obtained from the earlobe before and immediately after the exercise test to assess the LA levels.

### Measurements

Serum zonulin, intestinal fatty acid-binding protein (I-FABP), LPS, LBP, and interleukin 6 (IL-6) were measured using commercially available enzyme-linked immunosorbent assays (ELISAs; SunRed Biotechnology Company, Shanghai, China). The assay range was 0.25–70 ng/ml for zonulin, 0.3–80 ng/ml for I-FABP, 12–4000 endotoxin units (EU)/l for LPS, 0.2–60 µg/ml for LBP, and 1–300 ng/l for IL-6. Moreover, LA in capillary blood was measured immediately after sampling using a commercially available kit (Diaglobal, Berlin, Germany). The LA concentrations are presented as mmol/l.

### Statistical analysis

Statistical analysis was done using GraphPad Prism 9 (GraphPad Software, USA). Descriptive statistics such as the mean and standard deviation were used to identify patterns and trends. The Shapiro–Wilk test was carried out to examine whether the variables had a normal distribution. The Brown–Forsythe test was used to measure the equality of variances. One-way repeated-measures analysis of variance (ANOVA), with Tukey’s post hoc analysis, was used to assess differences in measured variables of the three assessment points (Pre, Post, and Recovery) for Tests I and II. A t-test was used to compare food intake, anthropometric characteristics, and 2000-m test outcomes (power, time, and LA) between Tests I and II. Cohen’s d was computed to determine the effect size. It was interpreted as small (0.2), moderate (0.5), or large (0.8) (Cohen, 1988). For correlation analysis, Pearson linear correlation coefficients were calculated. The significance of all statistical analyses was set at p ≤ 0.05. Based on a power analysis, all tests that yielded significant results had a power above 0.9, as calculated by G Power 3.1(G Power, (13).

## Results

### Food intake

There were no significant differences in the average intake of energy, protein, fat, carbohydrates, and fibre between Tests I and II (Table 3). We conducted the study during training camp at the Olympic Training Centres, where the athletes ate the same diet. Hence, the daily intake of calories, macronutrients, and fibre remained constant throughout the study period.

**Table 3:** Energy, protein, fat, carbohydrate, and fibre intake the day before Tests I and II.

	Energy [kcal]	Protein [g]	Fat [g]	Carbohydrates [g]	Fiber [g]
	**Test I**				
Mean	4504.90	239.10	165.20	528.80	31.00
Standard deviation	722.71	54.40	41.88	122.58	10.13
	**Test II**				
Mean	4413.20	187.14	151.32	601.00	35.68
Standard deviation	411.36	43.69	31.61	106.53	8.73

### 2000-m ergometer test

**Fig. 2:**
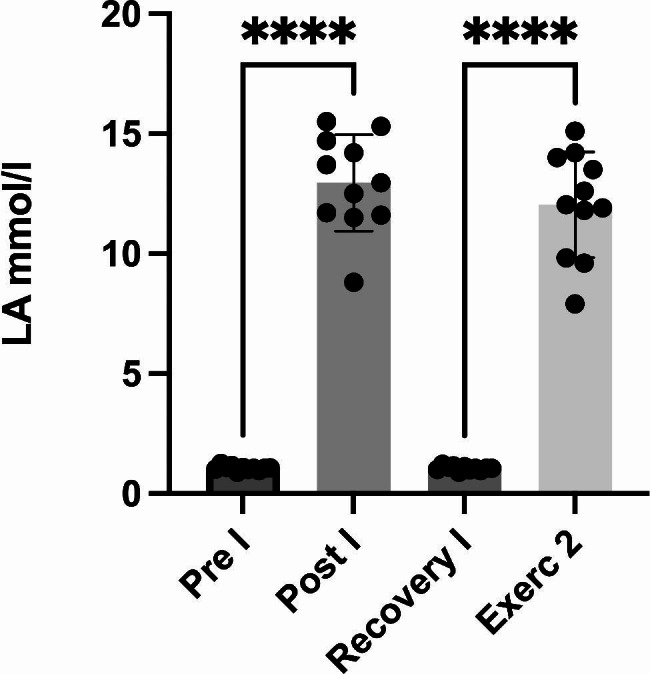
The lactic acid (LA) levels before and after the 2000-m ergometer test. The values are presented as mean ± standard deviation. *p < 0.05, **p < 0.01, ***p < 0.001, and****p < 0.0001

There were no significant differences between Tests I and II regarding the time to complete the test and the power (Test I: mean time = 6.09.00 min, SD = 7.05 s, and mean power = 450, SD = 26 W; Test II: mean time = 6.07.40, SD = 6.99 s and mean power = 444, SD = 25 W). The changes in the LA levels are shown in Fig. 2.

### Markers of gut injury and inflammation

**Fig3:**
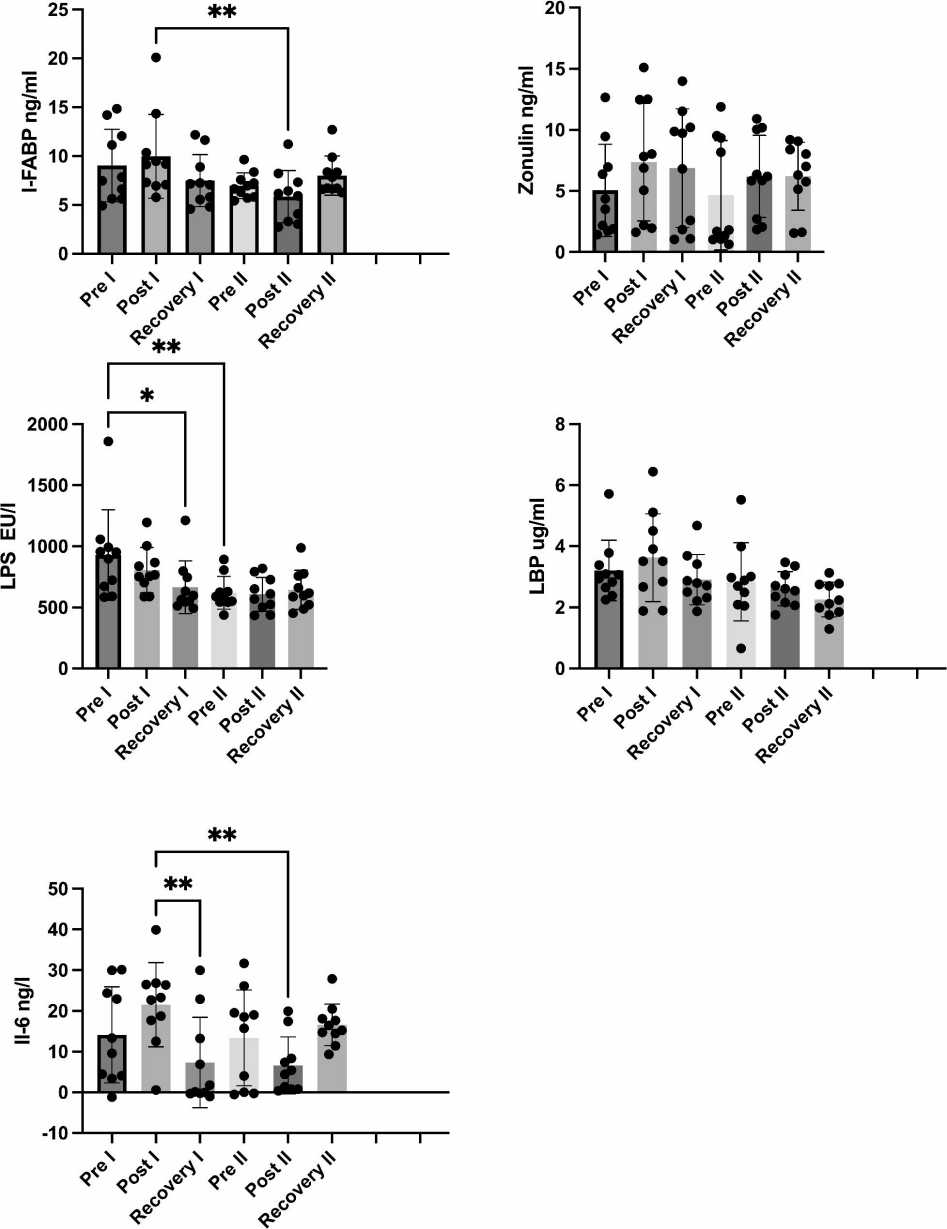
Differences between Tests I and II in intestinal fatty acid-binding protein (I-FABP), lipopolysaccharide binding protein (LBP), lipopolysaccharide (LPS), zonulin, and interleukin 6 (IL-6) at the pre-exercise (Pre), post-exercise (Post), and 1-hour post-exercise (Recovery) time points. The data are presented as the mean ± standard deviation. *p < 0.05, **p < 0.01, and ***p < 0.001

As shown in Fig. 3, in Tests I and II there were no differences in I-FABP between the three time points (Pre, Post, and Recovery). The level of I-FABP was significantly lower at the Post time for Test I compared with the Post time point for Test II (p = 0.0103, Cohen’s d = 1.11). There was no other differences between Tests I and II.

In Test I, there was no difference in the IL-6 level at the Post time point, and there was a decrease at the Recovery time point (p = 0.0064, Cohen’s d = 0.64). In Test II, there were no changes in the IL-6 levels between the three time points. The Post IL-6 level was significantly lower for Test II compared with the Post level for Test I (p = 0.0037, Cohen’s d = 1.98).

There were no changes in zonulin levels between the three time points for each test, and there were no differences between the tests.

There were no changes in LBP levels between the three time points for each test, and there were no differences between the tests. However, the difference in the LBP level between the Post times points for Tests I and II nearly reached significance (p = 0.058).

In Test I, LPS was not significantly different at the Post time point, but there was a significant difference between the Pre and Recovery time points (p = 0.0143, Cohen’s d = 0.9). In Test II, LPS was not significantly different at the Recovery time point. The Pre LPS level was significantly higher for Test I compared with Test II (p = 0.0026, Cohen’s d = 1.23).

**Fig. 4:**
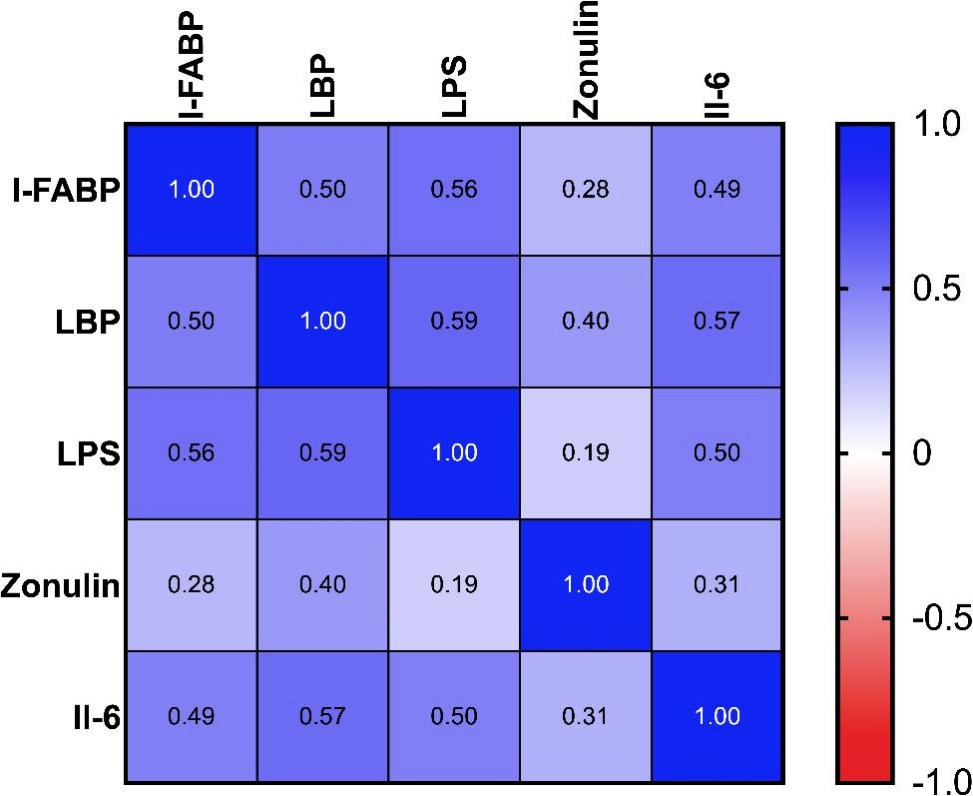
Pearson correlation matrix

We found a significant correlation between I-FABP and LPS (r = 0.56, p < 0.001), between I-FABP and LBP (r = 0.502, p < 0.001), between I-FABP and zonulin (r = 0.282, p = 0.029), and between I-FABP and IL-6 (r = 0.493, p < 0.0001). In addition, there was a significant correlation between LBP and LPS (r = 0.591, p < 0.001), between LBP and zonulin (r = 0.396, p = 0.0017), and between LBP and IL-6 (r = 0.557, p < 0.001). Finally, there was a significant correlation between IL-6 and LPS (r = 0.496, p < 0.0001). The correlation matrix is shown in Fig. 4.

## Discussion

The high-intensity 2000-m ergometer test was designed to replicate the physiological demands of competition in the different phases of the training cycle. In the present study, we found that the 2000-m ergometer test carried out during the preparatory and competitive phases did not influence markers of gut injury such as I-FABP and zonulin. We hypothesised that the mechanical strain of the rowing and the extremely high intensity of the test influences the gut barrier. However, we did not observe the expected changes, probably due to the short protocol time and/or small sample size. The outcomes confirmed that longer protocols are needed to observe significant changes.

The responses to investigated parameters to intense exercise differed between Tests I and II. In Test II, the I-FABP and IL-6 levels were lower at the Post time point (immediately after the exercise test) compared with the same time point in Test I. There was also a tendency for a significant difference in the LBP level (p = 0.058) at the Post time point. LBP and I-FABP are surrogate markers of intestinal epithelial cell injury [3]. Moreover, LBP is a marker of systemic endotoxemia, and IL-6 is connected to the inflammation-associated cytokine response. We should emphasise that IL-6 also has an anti-inflammatory effect in the context of exercise, but paired with the LBP it exerts an inflammatory effect [3].

In general, the I-FABP and LBP levels were lower in Test II (competitive phase) than in Test I (preparatory phase). The IL-6 response to exercise was also significantly lower for the Post time point in Test II compared with the same time point in Test I1. The observed differences in markers of gut injury may be an effect of the weaker inflammatory response during Test II, which was performed during the competitive phase.

During the preparatory phase, the intensity of the training load increases, which probably influences both gut leakage and endotoxemia. There are a few studies that have followed changes in gut permeability and injury markers in competitive elite athletes. Chantler et al. [14] observed elite rugby players (n = 19) during 6 weeks of preseason training. The exercise protocol test lasted 45 min and the gastrointestinal permeability markers tended to be lower after sports preparation, similarly to our results (but our test only lasted around 6 min) [14]. Chantler et al. [14] suggested that improved aerobic fitness may decrease the level of splanchnic hypoperfusion. We found that for Test II, the athletes finished the exercise test in a shorter time and achieved higher power (the change did not reach significance, but at the competitive elite level of sports performance, even 1 s makes a huge difference that might change the place in the ranking). This finding suggests that there was an improvement in exercise ability and demonstrated that the aerobic capacity may influence gut injury. Han et al. [15] showed that compared with non-elite athletes, the microbiome of elite rowers was dominated by the short chain fatty acid (SCFA)-producing bacteria, a finding that may confirm our results. Moreover, Keohane et al. [16] observed rowers during a 5000 km transoceanic race and found that microbial diversity increased throughout the event; there was also an increased abundance of butyrate-producing species and species associated with improved metabolic health [16]. Those results may confirm the adaptive capacity of the gut of competitive elite rowers.

In our study, the participants had higher baseline I-FABP levels for both tests (Test I = 9.033 ± 3.7 ng/ml; Test II = 6.96 ± 1.24 ng/ml) compared with other athletes [3, 5]. This observation has two explanations. First, it may be connected to training sessions before the test (Table 2). Second, rowing is a highly demanding sport, even for trained athletes. Elite rowers quickly achieve high energy expenditures and LA levels, which they must tolerate throughout the race [17]. LA levels above 8.7 mmol/l are connected to gastric ischaemia [18], one of the causes of gut permeability, and in our study, athletes reached even 12 mmol/l. Splanchnic hypoperfusion and subsequent gastrointestinal ischaemia are key factors that promote intestinal injury and hyperpermeability [8], which may explain high baseline I-FABP levels for Tests I and II.

The baseline LPS level was higher before Test I (928 ± 369,6 EU/l) compared with Test II (620 ± 133,9 EU/l). Our results confirm previous findings regarding the adaptive abilities of rowers, both before and after the exercise test. Baseline LPS values are important. Kahru et al. [19] observed that there is a difference in the LPS levels between runners who are symptomatic (767 ± 119 EU/l) and asymptomatic (567 ± 124 EU/l) for GIS occurrence [19]. The baseline Test I LPS level is notably above the symptomatic group from that study. Lim et al. [20] found that 14 days of increased training loads in trained endurance athletes reduced the plasma LPS levels at rest and 1.5 h after exercise but not during exercise [20]. The authors suggested that adapting the anti-LPS mechanisms to the different training loads may be effective during rest and recovery, but not during exercise. Regular exercise training can decrease circulating Toll-like receptor 4 (TLR-4) ligands, including saturated free-fatty acids (FFAs), extracellular heat shock proteins (HSPs), and LPS, which are known to promote proinflammatory cytokine production [21]. The changes in LPS levels are followed by IL-6 production, which was also blunted in Test II, and suggests the adaptive capacity of the rowers. The highest IL-6 level was after Test I, but the exercise programme appeared to reduce the IL-6 level. Similar results occurred in elite taekwondo athletes after 4 weeks of training [22]. Kaya [22] suggested that physical activity regulates immune responses by suppressing serum IL-6 levels [22].

The main findings of this study indicate that gut injury and inflammation vary over the season in competitive elite rowers. The results suggest that the participants adapted to increasing exercise loads during the preparatory phase. This adaptation could be related to a proper training schedule and recovery time.

## Conclusion

Although there were no significant changes in intestinal injury parameters in our study immediately after the 2000-m ergometer tests and after a 1-hour recovery period, the intestinal response was reduced over the training season. Changes in gut injury markers after extreme exercise tests may show that adaptive mechanisms have occurred. Even small changes in the parameters of the competitor may affect the deterioration of his or her well-being and the possibility of participating in the competition. It crucial that the gut of a competitive elite rower has the ability to adapt to high levels of performance.

Our study has a few limitations: the food intake was measured only 24 h before the test and during the study. It should be stressed that these athletes participated in training camp in Olympic Training Centres, where they ate the same diet (Table 3). However, future investigation should be extended to more detailed pre-test diet information.

## Data Availability

Due to ethical concerns, the datasets generated and analysed during the current study cannot be made openly available. However, they are available from the corresponding author upon reasonable request.

## Abbreviations

ATP: Adenosine triphosphate
FFAs: Free fatty acids
GIS: Gastrointestinal symptoms
HSPs: Heat shock proteins
I-FABP: Intestinal fatty acid binding protein
IL-6: Interleukin 6
LA: Lactic acid
LBP: Lipopolysaccharide-binding protein
LPS: Lipopolysaccharide
SCFA: Short-chain fatty acid
TLR-4: Toll-like receptor 4
$\dot V$O_2max_: Maximal oxygen uptake
Wmax: Maximal power output
